# Influence of Probiotic Administration in Canine Feed: A Comprehensive Review

**DOI:** 10.3390/vetsci12050449

**Published:** 2025-05-07

**Authors:** Niranjana Karukayil Gopalakrishnan, Manikantan Pappuswamy, Gomathy Meganathan, Sureshkumar Shanmugam, Karthika Pushparaj, Balamuralikrishnan Balasubramanian, In Ho Kim

**Affiliations:** 1 Department of Life Sciences, School of Sciences, Christ University, Bangalore 560029, Karnataka, India; 2 Department of Animal Resource and Science, Dankook University, Cheonan-si 31116, Chungnam, Republic of Korea; 3 Department of Zoology, School of Biosciences, Avinashilingam Institute for Home Science and Higher Education for Women, Coimbatore 641043, Tamil Nadu, India; 4 Department of Food Science and Biotechnology, College of Life Science, Sejong University, Seoul 05006, Republic of Korea

**Keywords:** canine probiotics, gut health, nutritional supplementation, immune function, digestive performance

## Abstract

Dogs are cherished companions, and in today’s world, pets are increasingly regarded as family members. Pet owners are placing growing emphasis on their animals’ health, particularly for dogs. Probiotics, which are living bacteria that benefit the host when given in sufficient quantities, have drawn a lot of interest in the veterinary nutrition community due to their beneficial effects on companion animals, including dogs. This study emphasizes the advantages of adding probiotics to canine diets in order to enhance the health of the gut flora and the technologies used to incorporate probiotics into canine feed. It looks at the best ways to deal with common dog health problems, highlighting probiotics as a helpful substitute for antibiotics, which can have serious adverse effects, encourage bacterial resistance, and disturb the gut’s microbial ecology, which is necessary for digesting. Such disruptions are linked to chronic inflammatory enteropathy and obesity in dogs. This paper also examines biotechnological advancements in probiotic incorporation methods in dog feed, aiming to optimize their health benefits. Probiotic feed supplements may thus represent a promising approach to advancing canine health care, providing a natural adjunct to conventional treatments and preventive measures.

## Simple Summary

Probiotics offer a natural approach to managing common canine health issues. Incorporating probiotics into canine diets supports a balanced gut microbiota, promoting overall digestive health and resilience in dogs, reducing reliance on antibiotics, which can disrupt gut flora, foster bacterial resistance, and cause adverse effects. Also, this review discussed technologies used to incorporate probiotics in dogs’ feed. Microencapsulation is a leading technology for incorporating probiotics into dog feed, as it effectively protects beneficial bacteria during processing and storage, ensuring optimal delivery and health benefits for dogs. Probiotics function gradually, supporting long-term health rather than offering an immediate cure. They are more effective in preventing diseases and maintaining a balanced gut environment than in providing rapid relief from existing conditions.

## 1. Introduction

**Figure 1:**
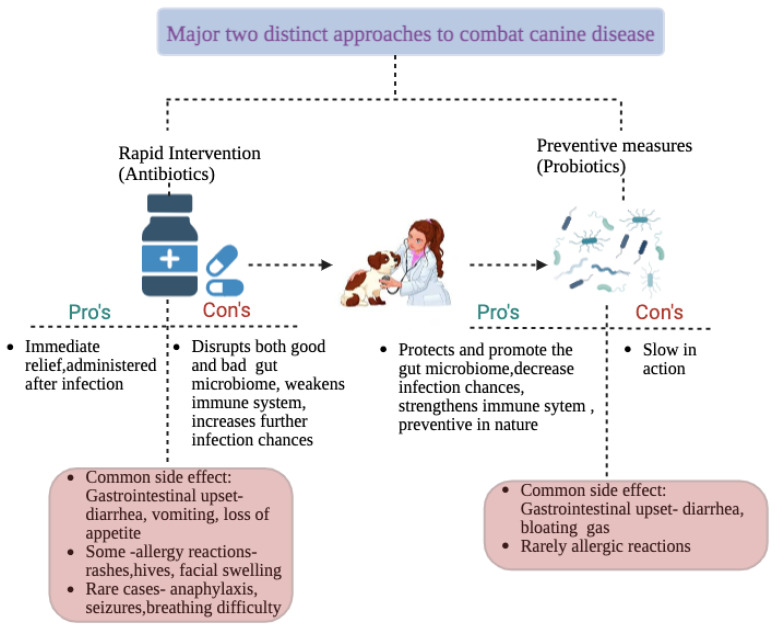
Two distinct approaches to combat canine diseases.

Innovative research aimed at enhancing the well-being and health of pets, especially dogs, who are cherished companions for many, is becoming more and more in demand in today’s society [1]. As the concept of pet ownership has evolved, dogs and cats are now more often regarded as family members, which has led to a greater focus on their overall health, especially gastrointestinal wellness [2]. This shift has sparked interest in novel strategies, such as incorporating probiotic supplements into canine diets [3]. More attention is currently being paid to gastrointestinal health and the function of gut bacteria in this system with the goal of improving pet welfare [4]. The use of probiotics and natural nutraceuticals, which are proven to have therapeutic effects for dogs, is made possible by this approach [5]. Probiotics are described as living bacteria that help the host’s health when ingested in sufficient amounts [6]. The intricate collection of bacteria known as the gut microbiota is vital to preserving general health because it supports metabolism, protects the intestinal barrier, and controls immunological responses [7]. Dysbiosis, a disorder characterized by imbalances in this microbial population, has been connected to a number of human and animal health issues [8]. In order to support intestinal microbiota balance and host health, probiotics have therefore been effectively included in functional foods and dietary supplements [9]. Broadly speaking, probiotics are living microorganisms (derived from fermented foods, gut bacteria, and other sources) that have been investigated in controlled studies, confirmed to offer health advantages, and their safety has been proved by testing [10]. Maternal antibodies, genetic factors, gut microbial makeup, and environmental stressors are some of the elements that influence the development of the intestinal immune system [11]. Microbes in the stomach are essential to the host’s immune system’s development. By altering the composition and activity of microbial communities, probiotic therapies could improve gut immunity [12]. Probiotics benefit the host in numerous ways, including improved digestion and nutrient absorption, strengthened immune responses, an increase in beneficial gut microbes, and inhibition of pathogenic bacteria [13]. Figure 1 depicts the distinct approaches of probiotics and antibiotics to combat canine disease. Additionally, they are crucial for treatment of a number of gastrointestinal conditions [14]. Important bacterial metabolites that promote host–microbiota interactions and have an influence on general health include short-chain fatty acids (SCFAs), secondary bile acids, and tryptophan metabolites [15]. Probiotics can improve the intestinal structure, optimize gut microbiota, decrease inflammation, and increase nutrient digestion, according to studies conducted on farm animals [16]. *Lactobacillus*, *Bifidobacterium*, *Enterococcus*, and *Saccharomyces* are common probiotic microorganisms found in functional foods and supplements [17]. Additionally, *Salmonella enteritidis*, *Staphylococcus aureus*, and *Escherichia coli* are among the frequent therapeutic bacteria that are killed by a type of *Lactobacillus* [8].

Probiotic supplementation has been increasingly recognized for its beneficial effects on gastrointestinal health and overall wellness in canines. Studies have demonstrated that probiotics can help maintain a balanced gut microbiota by promoting beneficial bacterial populations such as *Lactobacillus* and *Bifidobacterium*, which can suppress the growth of pathogenic organisms and reduce instances of diarrhea and gastrointestinal inflammation [17]. This microbial balance enhances nutrient absorption and digestion efficiency by supporting enzymatic activity and improving gut barrier integrity. Additionally, probiotics modulate the immune system by enhancing the mucosal immune response, increasing secretory IgA production, and reducing systemic inflammation, thereby contributing to improved immune resilience in dogs [18]. These cumulative effects underline the role of probiotics as a functional dietary intervention for promoting canine health.

Certain vitamins can be acquired by diet or gut flora, but dogs are unable to manufacture them on their own [18]. Essential vitamins, including folate and cobalamin, which are required for gastrointestinal stability, are thought to be abundant in the gut flora [19]. For example, a lack of folate can lower regulatory T (Treg) cells, a subgroup of T cells essential for immunological homeostasis, which can result in intestinal inflammation and poor gut health [20]. Probiotics have been used to treat gastrointestinal disorders, though their effectiveness depends on the specific bacterial strains [21]. While some probiotic bacteria produce vitamins, extended use of *E. faecium* SF68 has been associated with moderate hypocobalaminemia in healthy dogs, indicating that usage beyond 14 days might significantly reduce cobalamin levels [22]. Probiotic efficacy is largely determined by the particular microbial strain, dosage (CFU/day), and treatment duration [23]. The effects of probiotics might differ depending on the animal type, and they are often strain-specific [24]. This review focuses on the various effects of probiotic supplementation on gastrointestinal health, gut microbiota composition, impact of probiotics on nutrient absorption and digestion efficiency and analyzes the influence of probiotics on immune system and overall health status in canines.

## 2. Utilization of Probiotic Strains in Dog Food

### 2.1. Role of Lactobacillus Acidophilus in Gut Health

*Lactobacillus acidophilus* is a rod-shaped, Gram-positive bacterium that does not produce spores. Species within the *Lactobacillus* genus are widely recognized as beneficial intestinal probiotics that support and enhance gut microbiota [6]. *L. acidophilus* plays a significant role in supporting gut health in canines by promoting a balanced intestinal microbiota and enhancing gastrointestinal function. As a probiotic bacterium, *L. acidophilus* contributes to the production of lactic acid, which lowers the pH of the gut and creates an unfavorable environment for pathogenic microorganisms, thereby helping to prevent infections and gastrointestinal disturbances [6]. Certain dietary components known as prebiotics affect microbial populations and increase the intestines’ synthesis of SCFAs, which benefits the host’s health [6]. The spontaneous modification of the gut microbiota to promote beneficial health outcomes has garnered more attention recently. One such method is the feeding of probiotic *Lactobacilli* to animals. Species like *L. acidophilus* are found naturally in the gastrointestinal tracts of healthy dogs. Although individual variations exist, LB establishes itself in a dog’s gut shortly after birth, reaching a stable composition as the animal matures, where it primarily functions to inhibit the growth of harmful microorganisms [5].

**Figure 2:**
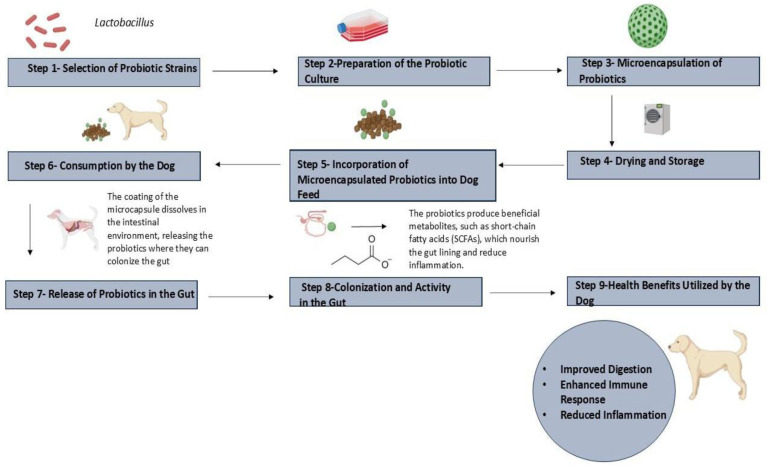
Steps in incorporation of probiotics in dog feed.

A higher frequency of proteobacteria (such as *Escherichia coli*) and a lower presence of Firmicutes in the intestines have been linked to inflammatory bowel disease (IBD) in both humans and dogs [5]. Research indicates that supplementing dog diets with *L. acidophilus* CECT 4529 improves their nutritional status [5]. Probiotic-enriched meals are associated with a decrease in clostridial organisms and an increase in fecal *Lactobacilli*. Along with decreased RBC fragility and decreased serum nitric oxide concentration, these diets have also been associated with increased levels of RBC, hematocrit (Hct), hemoglobin concentration, neutrophils, monocytes, and serum immunoglobulin G [8]. Probiotics also increased the frequency of defecations, dry matter content, and fecal consistency (*p* < 0.05). Fecal levels of culturable *Lactobacilli* and *Bifidobacteria* increased numerically, but these alterations fell short of statistical significance. Similarly, reductions in *C. perfringens* and *Escherichia* species did not achieve statistical significance. More stable bacterial populations were found using fluorescence in situ hybridization (FISH), which supports the idea that *L. acidophilus* DSM 13241 can help stabilize digestion mechanisms in dogs with non-specific food sensitivity [9]. The integration and role of probiotics in dog feed are illustrated in Figure 2.

Another important home for microorganisms is the oral cavity, which is why maintaining a balanced oral microbiome is crucial to prevent dental disorders caused by harmful bacteria. Tooth decay is due to acid production by *Streptococcus mutans*, and it can lead to damage and eventual tooth loss, wherein a healthy oral microbiome can effectively resist external influences, including colonization by harmful microorganisms. Research demonstrated the advantages of probiotics, especially *Lactobacillus* species, for the oral and gut microbiomes. Probiotics support oral health by directly or indirectly competing with pathogenic bacteria by displacing opportunistic pathogens, preventing pathogen attachment, disrupting bacterial biofilms, and exerting anti-inflammatory effects. Additionally, studies have demonstrated that dog-isolated strains of *L. acidophilus* had inhibitory effects on dental-caries-causing bacteria, with three strains showing very high suppression. Lactic acid bacteria (LAB) create microbial fractions, polysaccharides, and functional proteins during fermentation, all of which are beneficial to the host’s health [10,11,12].

*Lactobacillus acidophilus* is a beneficial bacterium commonly found in the gastrointestinal tract and widely used as a probiotic supplement. Its role in promoting gut health has been the subject of extensive research, highlighting several key benefits. Studies have demonstrated that *L. acidophilus* can strengthen the intestinal barrier function, which is crucial for protecting against pathogens and maintaining gut integrity. The *L. acidophilus* LA1 strain significantly increases the function of intestinal barrier cells, suggesting potential therapeutic applications for IBD and other intestinal inflammation disorders. *L. acidophilus* has been shown to influence electrolyte transport in the intestines [11]. Generally, it supports digestion by facilitating the breakdown of lactose and other carbohydrates, which can improve nutrient absorption, particularly in dogs with lactose intolerance or compromised digestive efficiency. Additionally, *Lactobacillus acidophilus* can help regulate the gut’s immune system by stimulating the production of immunoglobulins and enhancing the mucosal immune response, which plays a crucial role in defending against pathogens. Through these mechanisms, *Lactobacillus acidophilus* supports overall gut health, reduces inflammation, and improves the canine’s ability to absorb nutrients, contributing to better digestive wellness and immune function [11]. Research work indicated that this probiotic upregulates the expression and function of the Na^+^/H^+^ exchanger, which plays a vital role in sodium absorption. This modulation may contribute to its antidiarrheal properties, offering potential relief for individuals suffering from diarrhea [10]. Incorporation of *L. acidophilus* into the diet can positively alter the gut microbiota composition, promoting the growth of beneficial bacteria. This shift supported a healthy immune system by enhancing the gut’s defense mechanisms. Additionally, *L. acidophilus* may produce substances like lactic acid, which can inhibit the growth of harmful bacteria, further contributing to gut health [12].

### 2.2. Role of Saccharomyces boulardii in Immune System

The live yeast *Saccharomyces boulardii* is readily accessible as a food supplement and is frequently used as a probiotic. This yeast operates through multiple mechanisms targeting both the host’s health and harmful microorganisms by balancing intestinal microbiota, preventing pathogen colonization and infection in the mucosal lining, modulating immune responses locally and systemically, reinforcing gastrointestinal barrier integrity, and enhancing enzymatic activity to improve nutrient absorption [13]. Both dogs and cats may safely take this novel probiotic yeast [14], which has the potential to prevent and treat illnesses including IBS, antibiotic-associated diarrhea, *Clostridium* difficile infections, and non-specific diarrhea. Since gut microbes play a key role in preserving equilibrium across a number of body processes, including those pertaining to the brain, the heart, liver, kidneys, defense systems, and fat metabolism, a healthy gastrointestinal system is essential for general health. An imbalance in gut microbiota known as dysbiosis is frequently seen in healthy adult dogs and is frequently associated with aging; however, it can also happen to animals who live in stable conditions. These probiotics help maintain gut integrity by enhancing the production of SCFAs through fermentation, which supports the epithelial barrier and reduces intestinal permeability. By improving gut barrier function, *S. boulardii* limits the translocation of harmful pathogens, thus preventing systemic inflammation. Additionally, *S. boulardii* stimulates the innate immune system, enhancing the activity of macrophages and dendritic cells, which are key players in pathogen recognition and immune defense. This yeast also promotes the production of immunoglobulins, such as secretory IgA, which is crucial for mucosal immunity, thereby protecting the gastrointestinal tract from infections. Overall, *Saccharomyces boulardii* strengthens the immune response, supports gut health, and helps regulate the body’s inflammatory processes, making it a valuable supplement for maintaining immune system wellness [14].

Research has demonstrated numerous benefits of *S. boulardii* as a probiotic. It strengthens the intestinal barrier and supports tissue regeneration, making it a suitable alternative to antimicrobial treatments for dysbiosis. Studies show that *S. boulardii* supplementation significantly impacts zonulin and indole/skatole levels (*p* < 0.05 and *p* < 0.001, respectively), supporting its role in gut health [14]. Furthermore, analysis of indole/skatole and N-Methylhistamine levels showed no significant differences between groups, indicating that the supplement does not cause adverse effects [14]. Calprotectin, a major protein in neutrophil cytosol constituting about 60% of its protein content, is released into the intestinal lumen during inflammation, resulting in its presence in feces. *S. boulardii* supplementation has demonstrated positive benefits on indicators of inflammation, including calprotectin, immunological response reductions (IgA), and lowered markers of psychological stress (cortisol). Both calprotectin and IgA are recognized as non-invasive indicators of intestinal health in canines. Findings suggest that *S. boulardii* can help reduce intestinal inflammation and stress in animals [14,15]. According to clinical investigations, dogs with chronic enteropathies (CEs) treated with *S. boulardii* showed significant changes in their body condition score, frequency, consistency, and clinical activity index when compared to those given with a placebo. As a result, *S. boulardii* is seen to be a safe and efficient way to treat CEs in dogs, frequently producing greater symptom management than conventional therapies alone [16].

*Saccharomyces boulardii*, a probiotic yeast, has been studied for its potential effects on the immune system and overall gut health in dogs. Several studies indicated that supplementation with *S. boulardii* can lead to significant improvements in intestinal well-being and may modulate immune responses in canine subjects [14]. A research study involving dogs with CE demonstrated that administering *S. boulardii*, in addition to standard treatments, resulted in notable improvements. The treated dogs showed significant enhancements in the Canine Chronic Enteropathy Clinical Activity Index, including reduced stool frequency, improved stool consistency, and better body condition scores compared to the placebo group. Importantly, no short-term adverse effects were observed, suggesting that *S. boulardii* can be safely used in dogs with CEs [16]. Supplementation with *S. boulardii* has been associated with favorable changes in fecal biomarkers indicative of gut health. In a study with healthy adult dogs, S. boulardii administration led to a significant decrease in fecal calprotectin and IgA levels. These changes suggested an improvement in gut well-being and a reduction in intestinal inflammation. Additionally, a significant reduction in fecal cortisol levels was observed, indicating a potential reduction in stress among the dogs receiving the supplement [14]. Research has also reported the effects of *S. boulardii* on the gut microbiota composition in dogs. A study involving healthy adult American Staffordshire Terrier dogs found that supplementation with *S. boulardii* did not result in significant changes in the overall composition of the fecal microbiota and mycobiota. However, there was a notable increase in fecal IgA levels, highlighting an enhancement in mucosal immunity [17]. Further research indicates that *S. boulardii* supplementation in female dogs may act as a gut stabilizer during whelping and modulate the immunometabolic phenotype of their puppies. The study observed that bitches supplemented with *S. boulardii* exhibited increased plasma concentrations of interleukin-10 (IL-10) and transforming growth factor-beta (TGF-β), both associated with anti-inflammatory responses. After vaccination, puppies from supplemented bitches showed a significant decrease in the IL-8: IL-10 ratio, suggesting a balanced immune response and reduced inflammation [18].

## 3. Canine Chronic Inflammatory Enteropathy and Probiotics

In dogs, chronic inflammatory enteropathy (CIE) refers to a broad category of idiopathic conditions marked by persistent digestive (GI) symptoms. Food-responsive (FRE), antibiotic-responsive (ARE), immunosuppressive-responsive (IRE), and non-responsive (NRE) enteropathy are the four main disease phenotypes that have been discovered when traditional dietary therapies alone are not enough [19,24]. Probiotics have emerged as a promising adjunct therapy for managing CIE in dogs as they help restore the balance of beneficial gut microbiota, which is often disrupted in affected animals. Strains such as *Lactobacillus*, *Bifidobacterium*, and *Enterococcus* have been shown to reduce gastrointestinal inflammation, support intestinal barrier integrity, and modulate immune responses. These aid by enhancing the production of anti-inflammatory cytokines, improving the gut’s mucosal defense and preventing the overgrowth of harmful bacteria. By rebalancing the gut microbiota and supporting immune function, probiotics can help reduce the severity of symptoms and improve the overall health of dogs with CIE, offering a natural and beneficial complement to dietary management and medical treatments.

While these treatments often achieve high rates of clinical remission, repeated or long-term use of these medications requires careful consideration due to potential side effects. Studies have shown that antibiotics, in particular, negatively affect gut microbiota, reducing microbial diversity, balance, and species richness, which results in dysbiosis [19,20]. Furthermore, the development of multidrug-resistant infections in dogs is closely associated with the use of broad-spectrum antibiotics, posing major health hazards to both people and animals [19,20]. Table 1 shows the various diseases associated with dogs and the common usage of probiotics for their treatment. Fecal calgranulin C (S100A12) and calprotectin (CP) are indicators of GI inflammation in dogs with chronic enteropathy [13]. It has been demonstrated that the severity of the condition is correlated with elevated levels of fecal S100A12 and CP in dogs with CIE. One strategy for lowering the risk of NSAID-induced gastrointestinal problems is to take probiotics in addition to NSAIDs. Although previous research had demonstrated elevated counts of *Lactobacillus* in dogs with LAB, one study found no greater fecal levels of these bacteria in dogs receiving LAB compared to those in the placebo group. Although short-term NSAID usage has been demonstrated to impact human gut microbiota, this discrepancy may be because NSAID-induced microbiome alterations take longer to manifest [25].

For a microorganism to be classified as a probiotic, it must meet specific standards, such as remaining viable during production, storage, and passage through the GI tract, having no toxic or pathogenic effects, and providing measurable health benefits. To date, the most studied probiotic strains in dogs are Gram-positive bacteria, including *Bifidobacteria*, *Lactobacilli*, *Enterococci*, and *Bacilli* [19,21]. The phrase symbiotic describes the use of probiotics in conjunction with substrates that are specifically used by host microorganisms to promote the host’s health [19,22]. Probiotic effects may be broadly categorized into two primary mechanisms: immune function regulation and competitive inhibition against pathogenic microorganisms. In the first method, probiotics prevent harmful gastrointestinal bacteria from adhering to adhesion receptors on epithelial cells or in mucus. By competing for resources, generating antimicrobial compounds (such as bacteriocins and organic acids), and fortifying the intestinal barrier, probiotics also make the environment less favorable for pathogenic microorganisms [19,23].

By changing the gut microbiota, competing for adhesion sites on mucosal and epithelial surfaces, strengthening the gut membrane barrier, and modifying the body’s defenses to the host’s advantage, probiotics have been shown to have antagonistic effects on infections. A growing body of research suggests that probiotics interact with the host through pattern recognition receptors, such as nucleotide-binding oligomerization domain-like receptors and Toll-like receptors, which control key signaling pathways, such as nuclear factor-θB and mitogen-activated protein kinase, to alter immune activation and subsequent reactions [23]. One study provided a probiotic supplement containing *Lactobacillus casei*, *Lactobacillus plantarum* P-8, and *Bifidobacterium animalis* sub sp. *lactis* V9 in equal amounts (2 × 10^9^ CFU/g) at dosages of 2 g/day for young dogs, 4 g/day for active dogs, and 10 g/day for elderly dogs over a two-month period. This supplementation increased the levels of *Lactobacillus* sp. and *Faecalibacterium prausnitzii* while decreasing *Sutterella stercoricanisin* and *E. coli* in the feces of elderly dogs. Additionally, cytokine and antibody production were enhanced in the probiotic group. After two months, elderly dogs’ gut microbiota resembled that of younger dogs [27]. In another study, administering *Enterococcus faecium* EE3 (10^9^ CFU/mL) to healthy dogs over one week resulted in reduced fecal *Staphylococci* and *Pseudomonas* levels, with most dogs also showing lower total lipid levels and reduced cholesterol [37].

The European Commission recently authorized *Bacillus subtilis*, a rod-shaped, Gram-positive bacteria that forms spores as a gut flora stabilizer for dogs. Because spores’ durability and metabolic dormancy allow for longer storage periods and improved gut survival, *B. subtilis* C-3102’s capacity to generate spores gives it an edge over conventional probiotic microbes [24]. This resilience suggests that probiotics, such as *B. subtilis*, could be a practical approach for supporting canine health and mitigating disease risks. Though there are limited studies on *B. subtilis* in dogs, some findings indicate that it improves intestinal health by enhancing fecal quality and altering gut microbiota. Nutrient digestibility is a primary measure of dog diet quality and is linked to optimal GI health. Studies at Selçuk University found that diets containing *B. subtilis* improved dry matter digestibility in dogs [38]. Dogs fed a meal containing *B. subtilis* (C-3102) appeared to digest fat and nitrogen-free extracts more readily, according to another research. Additionally, elevated levels of RBC, WBC, and lymphocytes were noted, indicating immunological strengthening. According to this research, dogs with gastrointestinal infections benefit more from probiotics such as *B. subtilis* [38].

## 4. Obesity in Dogs and Probiotics

Obesity is defined as a condition of chronic excess body weight that results in health complications. It is a significant health issue affecting both humans and dogs, often leading to a range of secondary health issues, including reduced bacterial diversity in the gut. Subcutaneous fat mass, thickness, body weight, body condition score (BCS), and body fat percentage are all factors linked to obesity [39,40]. Numerous health issues, including chronic inflammation, a higher risk of diabetes mellitus, hypertension, degenerative orthopedic disorders, and reduced cardiac function, can be brought on by obesity [40]. Additionally, by decreasing the variety of the gut microbiota, obesity has a negative impact on gastrointestinal health. Serotonin, a neurotransmitter that reacts to food in the stomach, is frequently reduced in obese dogs. Reduced serotonin levels can slow colonic transit time, increasing fermentation time for undigested carbohydrates and fats, which subsequently alters the intestinal microbiome. Studies show marked differences between the gut microbiomes of lean and obese animals, with obese animals demonstrating lower bacterial diversity [39,40,41]. Probiotics have emerged as a promising adjunct therapy for managing CIE in dogs, as they help restore the balance of beneficial gut microbiota, which is often disrupted in affected animals. Strains such as *Lactobacillus*, *Bifidobacterium*, and *Enterococcus* have been shown to reduce gastrointestinal inflammation, support intestinal barrier integrity, and modulate immune responses. These aid by enhancing the production of anti-inflammatory cytokines, improving the gut’s mucosal defense and preventing the overgrowth of harmful bacteria. By rebalancing the gut microbiota and supporting immune function, probiotics can help reduce the severity of symptoms and improve the overall health of dogs with CIE, offering a natural and beneficial complement to dietary management and medical treatments.

The effect of probiotics in treating canine obesity has been the subject of several research studies. *Lactobacillus gasseri* BNR17 is a promising probiotic strain that has been shown to reduce body fat in various animals [39]. Research on the probiotic strains *Bifidobacterium lactis* IDCC 4301 and *Enterococcus faecium* IDCC 2102 has shown that they can decrease lipid accumulation and weight gain in obese dogs and hyperlipidemic caenorhabditis worms brought on by high-fat diets. Both strains helped reduce fat-related inflammation and hormone abnormalities. Additionally, they promoted the activation of beneficial LAB, including *Lactobacillaceae*, *Ruminococcaceae*, and S24-7, as well as pyruvate metabolism [41]. In particular, IDCC 4301 promoted the synthesis of short-chain fatty acids and carboxylic acids, which, in turn, promoted glycolysis and ATP generation. Healthy bacteria such as *Bifidobacterium, Enterococcus, Mogibacterium,* and *Clostridium* were found to be more prevalent in dogs supplemented with these probiotics. These bacteria improved the digestion of senior dogs, decreased diarrhea, decreased serum glucose and cholesterol levels, and decreased inflammation throughout the system [40,41]. While tyramine was lower in the obese group, several bacterial metabolites, including histamine, malonic acid, picolinic acid, methylsuccinic acid, and 5-hydroxy-L-tryptophan, were greater. Dogs on a high-fat diet (HFD) had lower increased metabolite levels after receiving probiotic therapy with *E. faecium* IDCC 2102 and *B. lactis* IDCC 4301 [41]. Probiotic treatments also changed the metabolic pathways of the HFD group, significantly improving the metabolism of amino acids, pyruvate, and glucose (glycolysis and gluconeogenesis). According to other research, probiotic treatments considerably decreased subcutaneous fat at the third lumbar vertebra, body weight, and BCS. Analysis of the microbiome diversity in obese dogs revealed enhanced metabolic processes for fat and carbohydrates, as well as increased microbial diversity [40].

Obesity-related diseases in dogs raise health care expenses, posing a financial burden on owners. Genetics, medical conditions (such as hypothyroidism), and an imbalance between caloric intake and energy are recognized risk factors for obesity in dogs [42]. Obesity is measured using a variety of methods, including body weight, electrical impedance, BCS, X-ray absorptiometry, ultrasound, and MRI. The BCS provides a subjective evaluation of muscle, ribs, spine, and subcutaneous and abdominal fat [42]. Obesity can result from gut microbiota imbalances brought on by an improper diet, which encourage the growth of dangerous bacteria [40]. On the other hand, obesity may be avoided or lessened by a balanced microbiome, which is supported by a nutritious diet. Research that looked at a probiotic formula including *Bifidobacterium breve* CBT BR3 and *Lactiplantibacillus plantarum* CBT LP3 in obese dogs found that the treatment group did not experience any adverse effects like vomiting or diarrhea. Body weight and BCS significantly decreased in dogs given this probiotic combination [43]. Obese dogs have a comparable blood lipid profile to obese people, with lower levels of high-density lipoprotein (HDL) and higher levels of triglycerides (TG), total cholesterol (TC), and low-density lipoprotein (LDL). It is noteworthy that the gut microbiota interacts significantly with the liver, brain, kidneys, bones, and heart, among other organs. The makeup of the gut microbiota specifically influences the gut–liver axis in a variety of liver diseases, including alcoholic liver disease, non-alcoholic fatty liver disease, and chronic hepatitis B and C. A healthier gut microbiota may improve many liver disorders. Studies have shown a significant reduction in serum aspartate aminotransferase (AST), alanine aminotransferase (ALT), and gamma-glutamyl transferase (GGT) levels with probiotic treatment [44].

## 5. Atopic Dermatitis and Probiotics

**Figure 3:**
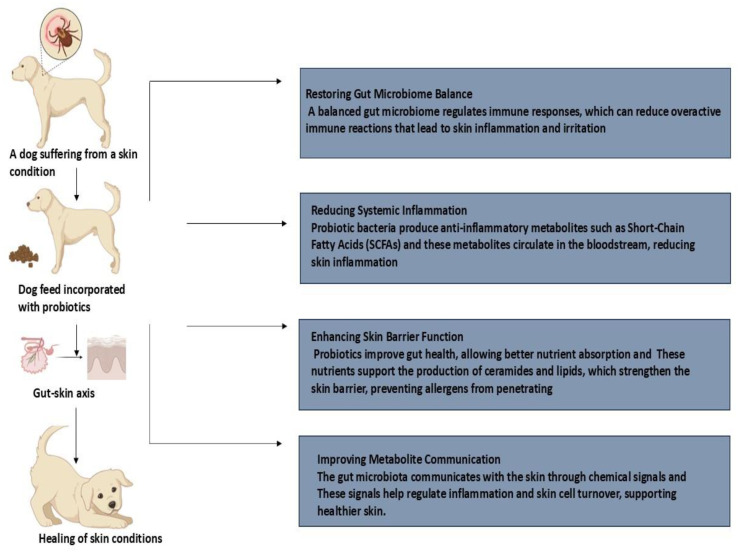
Probiotics enhance the gut–skin axis, aiding in atopic dermatitis treatment.

Exposure to different environmental allergens is associated with the development of canine atopic dermatitis (AD), a genetically predisposed allergic skin disorder that causes chronic itching in dogs. The development of immunoglobulin E (IgE) antibodies specific to allergens and identifiable clinical symptoms are common manifestations of this illness [45]. Several approaches, such as allergen avoidance, anti-inflammatory drug usage, and allergen-specific immunotherapy, have been proposed to treat the clinical signs of canine AD. Anti-inflammatory and anti-itch drugs, including systemic and topical glucocorticoids, ciclosporin, and oclacitinib, are the mainstay of treatment for canine AD. However, side effects have been linked to long-term or high-dose usage of these drugs [45]. Probiotics offer an alternative by modulating the immune response, potentially providing a protective effect against atopic dermatitis (Figure 3). Probiotics can reduce allergic reactions by balancing the Th1/Th2 immune system by reducing Th2 responses in favor of Th1. Th1 activity, which is essential for preserving an allergic phenotype, is also inhibited by Th2 cytokine response activation, mostly through interferon (IFN)-γ. The transcription factors T-bet (Th1) and GATA-3 (Th2) affect the stability of the Th1/Th2 equilibrium [46].

Studies indicate that probiotics, as adjunct treatments with specific microorganisms, may be effective in managing canine AD. A study demonstrated that oral administration of *Bifidobacterium longum* (5 × 10^10^ CFU/day) to dogs with AD over 12 weeks resulted in improvements in skin lesion severity, measured by the Canine Atopic Dermatitis Extent and Severity Index (CADESI) score. The findings revealed that *B. longum* administration progressively lowered CADESI scores compared to baseline levels, indicating that *B. longum* may be beneficial for improving skin lesions in dogs with AD [47].

## 6. Technologies Used to Incorporate Probiotics in Dog Feed to Ensure Maximum Effectiveness

Probiotics must survive in the canine digestive system in order to provide their health advantages. By improving the immunological and digestive systems of both dogs and cats, probiotics, also known as direct-fed microbials, are promoted as functional additives that raise the nutritional content of pet meals. Each probiotic strain’s specific metabolic characteristics and, most importantly, the number of live cells that reach the host’s large intestine dictate its therapeutic effects [48,49]. Incorporating probiotics into dog feed to ensure maximum effectiveness requires advanced technologies that protect the viability and stability of probiotic strains during storage, digestion, and transit through the gastrointestinal tract (e.g., microencapsulation and synbiotics). The goal of pet food production is to increase product safety and prolong shelf life. Probiotics must, however, endure a number of physical difficulties while being produced and stored. Species, strain, food type, pH, and interactions with other microbes are some of the variables that affect the addition of probiotics to diet. The resilience of each bacterial species under processing circumstances is determined by its unique metabolic requirements, growth rate, proteolytic activity, and survival strategies. For instance, the *Lactobacillus* genus thrives between 2 and 53 °C, with an optimal range of 30–40 °C, and prefers mildly acidic to neutral pH values, ideally between 5.5 and 6.2 [50]. In order to optimize the advantages of probiotic-enriched dog food, contemporary biotechnological techniques (Table 2) have been developed to extend its shelf life while preserving probiotic viability, efficacy, and safety during manufacture, storage, and digestion [51].

**Figure 4:**
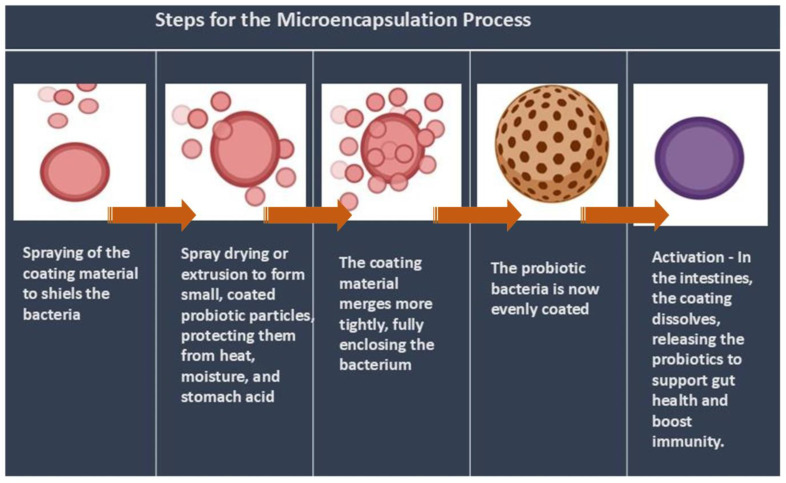
Steps in microencapsulation process.

A physicochemical process called microencapsulation enhances the functioning of active substances or cells by encasing them in a protective substance (Figure 4). Protecting probiotics from severe gastrointestinal conditions is the main goal of microencapsulation [52]. However, as the shelf life of probiotic meals is primarily determined by the pace at which viable probiotics diminish in the product, the impact of the food matrix and processing conditions on the survival of microencapsulated probiotic cells must be taken into account. For probiotics that are meant to colonize the gut and provide health advantages, microencapsulation has become a useful method for increasing the survivability of the bacteria in food and the intestines [49]. In order to increase probiotic life, a study investigated microencapsulation employing alginate, both by itself and in combination with goat milk. According to the results, alginate-goat milk microcapsules had the best encapsulation effectiveness and offered the best defense against LAB strains under all circumstances, including pasteurization. Viable cell counts surpassed 6 log CFU/g even after pasteurization, indicating the effectiveness of alginate-goat milk microcapsules for probiotic preservation [53].

This process converts ice directly to vapor, producing a dry substance. Freeze-drying preserves a large number of viable probiotics at low temperatures for extended periods and maintains the product’s appearance, quality, and active ingredients [52]. Extrusion cooking is the dominant technology in commercial pet food production, accounting for the majority of the pet food market. Extruded foods are nutrient-rich, flavorful, and shelf-stable, made using a continuous, high-throughput process. Extrusion is a high-temperature, short-duration, high-shear technique where pre-conditioned raw ingredients are conveyed through a barrel by a rotating screw, forced through a die, resulting in vapor flash-off and product expansion. This process is crucial for spore-forming microorganisms as it ensures their survival during extrusion while effectively eliminating pathogenic cells [50]. Thermal processing presents one of the greatest challenges for probiotics in food products. Most pet foods undergo some level of cooking or commercial sterilization to extend shelf life and eliminate pathogenic microorganisms or their toxins. However, while thermal processing is effective at reducing microbial activity, it can hinder the inclusion of probiotics. Due to dehydration and heat inactivation, spray-drying-a popular and economical microencapsulation method for probiotics-can jeopardize cell viability. To lower cell mortality, protective substances such as trehalose, adonitol, acacia, milk solids not fat, granular starch, and prebiotics can be added. High probiotic viability is a primary emphasis, and non-dairy spray-dried probiotic powders are in demand because of their stability, affordability, ease of use, and ease of incorporation into a variety of diets [50]. Freeze-drying, another method, involves freezing the material and sublimating moisture under vacuum.

**Table 2:** Technologies for integrating probiotics into dog feed and their benefits.

Technology Used	Benefits	Reference
Microencapsulation	Enhancing the survival of probiotic cells through gastrointestinal digestion and boosting their stability.	[49]
Edible Films and Coatings Functionalization by Probiotic Incorporation	Edible coatings and films provide an eco-friendlier and consumer-oriented alternative to conventional food packaging.	[54]
Freeze-Drying	Compared to other drying techniques, freeze-drying probiotics uses less energy while maintaining their original physical and chemical properties.	[52]
Fermentation Technologies	Continuous culture and immobilized cell systems are two examples of fermentation technologies that may increase the effectiveness of probiotic strains, improve gut function, increase the number of strains accessible, and expand the uses of products.	[55]
Encapsulation with Liposomes	It forms a protective barrier around the bacteria, shielding them while permitting small molecules to pass through.	[56]

## 7. Conclusions

Dogs are treasured companions, and in today’s world, there is an increasing emphasis on improving their health and well-being. The microbiome communicates with tissues and organs in both directions in an effort to maintain homeostasis as the microbiome is a crucial immune system regulator. As a result, skin and/or gut microbiome dysbiosis is linked to a changed immune response, which encourages the emergence of skin conditions like psoriasis and atopic dermatitis. By promoting gut microbial balance and lowering the risk of chronic diseases including obesity and inflammatory enteropathies, probiotics provide a viable preventative approach to improving canine health. While probiotics are not a quick fix, they serve as an effective, long-term approach to maintaining wellness and improving body condition in dogs. The observed benefits in body weight, body condition, and fecal health after prolonged probiotic use underscore their potential as part of a preventive regimen rather than a rapid cure. The stability and effectiveness of probiotics in pet meals have been enhanced by recent developments in biotechnological techniques, particularly microencapsulation, which increases the viability of these beneficial microbes throughout processing and storage. Combining probiotics with prebiotics or specific proteins like zonulin further enhances microbial stability and gut health, introducing an innovative approach for fostering intestinal health and overall well-being in dogs. Next-generation sequencing technologies now provide unprecedented insights into host–microbe interactions, offering new avenues for targeted therapeutic interventions. Despite significant advances, research on probiotics in companion animals still lags behind studies in human health, highlighting an urgent need for more comprehensive investigations in this field. Expanding research on canine models will not only deepen our understanding of probiotic functions beyond gut health but also open pathways for tailored probiotic formulations. This continued research could ultimately refine therapeutic strategies, leading to improved quality of life and longevity for our canine companions.

## Figures and Tables

**Table 1:** List of diseases in dogs and the probiotics commonly used for treatment or management.

Name of the Diseases	Symptoms	Probiotic Used	Reference
African trypanosomiasis	When trypanosomes penetrate the epidermal barrier, a localized inflammatory response known as chancre development is triggered.	*Lactobacillus reuteri*	[26]
Colitis	Large-bowel diarrhea is marked by symptoms such as the presence of mucus in the stool, fresh blood (hematochezia), a frequent urge to defecate (tenesmus), and sometimes discomfort or pain during bowel movements.	*Lactobacillus acidophilus*	[27]
Exocrine Pancreatic Insufficiency	This disorder is brought on by insufficient pancreatic digesting enzymes in the small intestine. Symptoms typically include weight loss, large, soft stools, and an increased appetite.	*Lactobacillus reuteri*	[28]
Malassezia dermatitis	They are usually marked by redness (erythema), flaking (scaling), and/or oily discharge. In long-term cases, thickening of the skin (lichenification) and darkening of the skin (hyperpigmentation) may also occur.	*Lacticaseibacillus paracasei*	[29]
Seborrheic dermatitis	Small, thin patches coated with oily scales are the hallmark of seborrheic dermatitis, which is most frequently observed in regions including the scalp, face, chest, back, and skin folds that have a high density of sebaceous glands.	*Lactobacillus crispatus*	[30,31]
Stress-Induced Dermatitis	Comfort-seeking, grooming, less trainability, fear- and anxiety-related behaviors, aggression, excitability, and attention-seeking.	*Lactobacillus plantarum*	[32,33]
Canine Chronic Inflammatory Enteropathy	Vomiting and diarrhea.	*Ascophyllum nodosum* and *Bacillus subtilis C-3102*	[24]
Halitosis	Bad breath.	*Lactobacillus acidophilus*	[11]
Periodontal Disease	Gum inflammation and infection, foul breath, and tooth loss are among the symptoms.	*Lactobacillus acidophilus*	[11,34]
Candidiasis	Debris accumulation in one or both ears that is yellow, brown, or black is one of the signs. An excessive amount of head shaking and scratching of the ears or other body parts.	*Saccharomyces boulardii*	[35,36]

## Data Availability

The data presented in this study are available on request from the corresponding authors.
